# Reply to Douxfils, J.; Foidart, J.-M. Critical Considerations in the Interpretation of Bone Turnover Marker Data in Hormonal Contraceptive Users. Comment on “Tassi et al. Hormonal Contraception and Bone Metabolism: Emerging Evidence from a Systematic Review and Meta-Analysis of Studies on Post-Pubertal and Reproductive-Age Women. *Pharmaceuticals* 2025, *18*, 61”

**DOI:** 10.3390/ph18091402

**Published:** 2025-09-18

**Authors:** Alice Tassi, Ambrogio P. Londero, Anjeza Xholli, Giulia Lanzolla, Serena Bertozzi, Luca Savelli, Federico Prefumo, Angelo Cagnacci

**Affiliations:** 1 Obstetrics and Gynecology Unit, Morgagni-Pierantoni Hospital, 47121 Forlì, Italy; 2 Department of Neuroscience, Rehabilitation, Ophthalmology, Genetics, Maternal and Infant Health, University of Genoa, 16132 Genova, Italy; 3 Obstetrics and Gynecology Unit, IRCCS Istituto Giannina Gaslini, 16147 Genova, Italy; 4 Academic Unit of Obstetrics and Gynecology, IRCCS Ospedale San Martino, 16132 Genoa, Italy; 5 Endocrinology Unit, Department of Medical Science and Public Health, University of Cagliari, University Hospital of Cagliari, 09124 Cagliari, Italy; 6 Breast Unit, University Hospital of Udine, 33100 Udine, Italy; 7 Dipartimento di Scienze Mediche e Chirurgiche (DIMEC), University of Bologna, 40138 Bologna, Italy

We express our gratitude to Douxfils and Foidart for their interest in our meta-analysis regarding bone marker modifications associated with contraceptive use in post-pubertal and reproductive-age women [1]. We appreciate the opportunity to respond to the issues presented in their commentary [2].

When assessing the effects of hormonal contraception on bone health, outcomes can be ranked by clinical relevance: serum estradiol, bone turnover markers (BTMs), bone mineral density (BMD), and fractures. Fractures represent the most definitive outcome; however, they pose significant challenges in assessment. Fracture data generally originate from extensive cohort studies in which hormonal contraception is frequently not analyzed by its individual components. Several studies indicate that the use of combined oral contraceptives (COCs) among diverse populations, including adolescents, is linked to a modest and inconsistent elevation in fracture risk [3,4,5,6,7,8,9]. However, these studies frequently suffer from confounding factors due to insufficient stratification of the androgenicity of the progestogen component and the inclusion of women exhibiting symptoms of hyperandrogenicity.

In contrast, initial research indicated that formulations containing higher doses of ethinylestradiol (EE) in conjunction with androgenic progestins resulted in increased bone density in young women when compared to control groups [10]. This finding aligns with the androgen-driven osteoblastic activity noted in conditions such as Polycystic Ovary Syndrome (PCOS), where increased endogenous androgens correlate with a higher peak bone mineral density (BMD) [11].

According to Douxfils and Foidart, BMD necessitates an extended duration to exhibit substantial changes. Given that the coefficient of variation for DEXA is 0.5–1% [12], a study period of at least two years is required to document even minor effects, a duration that studies on different contraceptive combinations rarely achieve. Furthermore, BTMs can detect aspects of skeletal fragility that DEXA cannot. There is evidence that bone fractures in premenopausal women are linked to BTMs, which are independent predictors of bone fracture [13,14]. In contrast, in the same evidence, BMD did not distinguish between fracture patients and controls [13,14]. These findings support the use of BTMs as remodeling indicators in settings where DEXA-detectable changes require longer follow-up, while emphasizing that BTMs are not BMD surrogates [13,14]. This review examines the differential effects of combined hormonal contraceptive components on bone metabolism, exclusively incorporating prospective studies, 11 of which were randomized, involving healthy women. BTMs were selected as the primary endpoint because most prospective studies with uniform therapy arms are not primarily intended to evaluate bone outcomes. As a result, BTMs are frequently utilized, particularly for short-term follow-up in young, healthy women, since these studies lack sufficient power and duration to identify changes in BMD or fracture incidence.

Although a single BTM value does not directly indicate BMD, our study assessed the changes in BTMs over a specified duration. The commentary presents evidence suggesting that alterations in bone formation markers do not consistently correlate with treatment-induced changes in bone mineral density (BMD) [15,16,17,18]. Nevertheless, it does not acknowledge that these sources indicate a consistent association between modifications in bone resorption markers and changes in bone mineral density during anti-resorptive therapies [15,16,17]. We aimed to provide a comprehensive evaluation of the net effect on bone metabolism by analyzing changes in formation and resorption markers relative to baseline.

The commentary mistakenly presumes that our comments were based on the assumption that equivalent percentage changes in formation and resorption markers correspond to stable bone mineral density (BMD). Our methodology differed. The natural variation in BTMs observed in untreated control groups served as our reference point. The primary inquiry was as follows: which CHC formulations minimally impact bone turnover in comparison to the anticipated physiological changes over time? Consequently, the formulations exhibiting marker shifts that closely aligned with those of the control groups were deemed the least disruptive to normal bone equilibrium. The findings from our meta-regression analyses support a robust physio-pathological rationale, highlighting the specific effects of various estrogen doses and compounds in conjunction with progestins exhibiting different androgenic characteristics [1].

To synthesize data from studies with varying measurements of BTMs, we calculate the standardized mean change (SMC) for each result to facilitate comparison [1]. Essentially, the measure was derived by calculating the difference between follow-up and baseline values, dividing by the pooled standard deviation. This method standardized effect sizes across various markers and assays, enabling the aggregation of results on a unified scale. In addition, to account for confounding factors and variability across studies, we advanced beyond SMC by employing multivariable meta-regression [1]. In the meta-regression, we accounted for study-level differences, which serve as a proxy for methodological heterogeneity, including assay platform, sampling timing, and participant composition [1]. Additionally, we considered follow-up duration, age, recognizing the limitation of using the maximum reported age in the absence of finer stratification, and pharmacologic moderators that reflect progestin androgenicity and the estrogenic effect on SHBG [1]. Sensitivity analyses were conducted, including stratification by age and leave-one-out procedures for strata with three or more studies [1]. We concur that these strategies cannot completely eliminate all residual confounding; thus, we have explicitly addressed heterogeneity across studies in the Limitations section. The direction and magnitude of effects were consistent across both univariate and multivariate models, demonstrating the robustness of our pooled estimates; however, careful interpretation remains necessary [1].

The commentary accurately observes that BTMs do not serve as established predictors of fractures in young women. The aim was not to establish a correlation between BTMs and fracture risk, given the low prevalence of this outcome in the demographic studied. This study aimed to investigate the effects of various contraceptive formulations on the equilibrium between bone formation and resorption. This analysis, though perceived as limited by commentators, offers significant insights into changes in bone metabolism that, if persistent, may impact bone mass and subsequently influence osteoporosis risk in later life. Studies evaluating fractures have demonstrated that both bone turnover markers (BTMs) and bone mineral density (BMD) are independently associated with fracture risk [15,16,17].

Progestin-only contraception (POC) was not the primary focus of our meta-analysis. However, we must challenge the assertion that all POCs induce bone loss and universally require estrogen for bone preservation. Depot medroxyprogesterone acetate (Provera) is recognized for its association with bone loss; however, this effect has not been established for other progestins. We agree with Douxfils and Foidart that the FDA warning regarding drospirenone indicates a potential adverse effect, but that was not a definitive confirmation of harm [19]. Currently, there are no published studies documenting BTM or BMD outcomes for drospirenone-only or desogestrel-only pills. A recent consensus suggests that women over 40 years of fertile age requiring contraception lack direct BMD data for progestin-only pills [20]. Instead, the consensus relied on estrogen levels as proxies for bone effects, underscoring the evidence gap concerning bone endpoints associated with progestin-only contraceptives [20].

Research on POCs that include drospirenone, norethindrone, or desogestrel shows that estradiol levels are sustained within the follicular phase range, deemed adequate for the preservation of bone mass [21,22]. POCs, when administered at doses that inhibit ovulation, sustain estradiol levels comparable to baseline and those observed with combined hormonal treatment [23,24,25,26].

The assertion that the inclusion of estrogen with progestin is universally essential for bone health fails to consider multiple limitations. The evidence provided from adolescents with estrogen insufficiency pertains to a distinct clinical context: the induction of puberty in individuals who lack endogenous estrogen production [27]. These studies often use replacement regimens such as transdermal estradiol or conjugated equine estrogens, and thus focus on an overall therapeutic effect rather than on bone or a contraceptive setting [27]. By contrast, our review was purposefully limited to combination hormonal contraceptives in healthy post-pubertal and reproductive-age women, as determined by our predefined inclusion criteria. By combining these disparate samples, the generalizability of the findings may be overstated. We agree that estradiol demonstrates a superior profile compared to ethynyl estradiol in terms of bone metabolism, as reported in our analysis [1]. Currently, there is a lack of data regarding estradiol contraceptive combinations for individuals under 21 years, as well as insufficient information on other natural estrogen combinations or POCs within this age group in the healthy population. Moreover, the physiological consequences of exogenous estrogens differ from those of endogenous secretion. Exogenous estrogens inhibit gonadotropin secretion, leading to a decrease in endogenous ovarian estrogen synthesis [23]. Exogenous estrogens replace this physiological signal, carrying distinct metabolic implications. Oral administration of estradiol leads to the significant production of estrone, a weaker estrogen that may compete with other estrogens at the receptor level. Oral estrogens enhance hepatic production of proteins such as sex hormone-binding globulin (SHBG) and insulin-like growth factor 1-binding proteins (IGFBP-1), while simultaneously reducing IGF-I synthesis [23,28]. The observed dose- and molecule-dependent reduction in free androgens and IGF-I stimulation of bone is a significant factor to consider. The effect on SHBG is dose-dependent, being most pronounced with ethinylestradiol, lower with estradiol, and estetrol [23,29]. Estetrol possesses a distinctive receptor profile; it has a positive effect on bone in a rat osteoporosis model [30,31], but its precise impact on bone of women, particularly of those women in reproductive age, remains inadequately characterized, given its reported capability to antagonize some estradiol-mediated effect [1,12,32,33]. The hypothesis that the addition of estrogen universally spares bone requires further empirical validation.

In conclusion, we acknowledge the valuable feedback provided by Douxfils and Foidart. Our meta-analysis did not aim to assess fracture risk or long-term changes in bone mineral density, due to the lack of necessary long-term, stratified prospective data at present. Instead, our analysis offers the most reliable evidence on the short-term effects of different CHC formulations on the balance between bone formation and resorption. By comparing these shifts to the physiological changes in control groups, we provide a nuanced understanding of how various estrogen and progestin combinations affect bone turnover.

Although we agree that these findings cannot be directly applied to lifetime fracture risk, they offer important insights for clinicians in choosing formulations that are likely to minimize disruption to bone metabolism in young women. Further research, especially long-term studies on bone mineral density and fractures, is necessary.
